# Burden of Oral Health-Related Quality of Life Among Pediatric Cancer Patients: Evidence from Saudi Arabia

**DOI:** 10.3290/j.ohpd.c_2571

**Published:** 2026-03-13

**Authors:** Shaikha Aldukhail, Hadeel Jiffry, Aseel Salem, Fotoon AlOtaibi, Hazar AlHarbi

**Affiliations:** a Shaikha Aldukhail Associate Professor, Department of Preventive Dental Sciences, College of Dentistry, Princess Nourah bint Abdulrahman University, Riyadh, Saudi Arabia. Conceptualization, methodology development, data analysis and visualization, wrote the original draft, reviewed and revised the manuscript.; b Hadeel Jiffry Dentist, College of Dentistry, Princess Nourah bint Abdulrahman University, Riyadh, Saudi Arabia. Conceptualization, methodology development, data collection, wrote the original draft, reviewed the manuscript.; c Aseel Salem Dentist, College of Dentistry, Princess Nourah bint Abdulrahman University, Riyadh, Saudi Arabia. Conceptualization, methodology development, data collection, wrote the original draft, reviewed the manuscript.; d Fotoon AlOtaibi Dentist, College of Dentistry, Princess Nourah bint Abdulrahman University, Riyadh, Saudi Arabia. Conceptualization, methodology development, data collection, wrote the original draft, reviewed the manuscript.; e Hazar AlHarbi Assistant Professor, Department of Basic Dental Sciences, College of Dentistry, Princess Nourah bint Abdulrahman University, Riyadh, Saudi Arabia. Conceptualization, reviewd and revised manuscript.

**Keywords:** dental care access, OHIP-14, oral health practices, oral health–related quality of life, pediatric cancer, supportive care in oncology.

## Abstract

**Purpose:**

Oral health-related quality of life (OHRQoL) is a critical dimension of well-being, particularly for pediatric cancer patients. Because limited evidence is available from Saudi Arabia regarding OHRQoL in this vulnerable population, this study aimed to assess and explore the factors influencing OHRQoL among pediatric cancer patients and survivors in Saudi Arabia using the OHIP-14 questionnaire, and to describe self-reported oral health practices, access to dental care, and related behaviors in this population.

**Materials and Methods:**

This hospital-based cross-sectional study was conducted among 100 pediatric cancer patients (aged 1–12 years) at King Faisal Specialist Hospital and Research Center, Riyadh, between February and April 2024. Data were collected using a structured questionnaire that captured sociodemographic characteristics, oral health practices, access to dental care, and responses to the validated OHIP-14 scale. Each OHIP-14 item was rated on a 5-point Likert scale, with higher scores indicating poorer OHRQoL. Descriptive and inferential statistical analyses, including chi-squared tests, were performed, and 95% confidence intervals were calculated.

**Results:**

Leukemia and related hematologic malignancies were most common (42%), followed by lymphomas (12%). Chemotherapy alone was the predominant treatment (44%). Over half of participants (52%) had OHIP-14 scores ≥3, indicating poor OHRQoL, most notably in the physical pain (74%) and physical disability (52%) domains. The most frequent severe impacts were “pain in the mouth” (25%), “difficulty eating” (23%), and “interrupted meals” (21%). Half of the patients (50%) had not visited a dentist within the past year, and 30% reported not brushing daily. Chi-squared analyses revealed statistically significant associations between access to dental care and several sociodemographic and clinical factors (p < 0.001 for household size, parental education, job, cancer diagnosis, and treatment). Similarly, high OHIP-14 scores were statistically significantly related to age, parental education, and cancer- and treatment-related variables.

**Conclusions:**

Pediatric cancer patients in this study demonstrated considerable OHRQoL impairments, particularly in relation to pain and functional limitations. The findings underscore the importance of integrating routine oral health assessment and supportive dental care into pediatric oncology services in Saudi Arabia.

Oral health is a fundamental component of overall well-being, influencing essential aspects of daily life such as function, appearance, speech, and psychosocial health.^8^ According to the World Health Organization (WHO), quality of life is defined as “individuals’ perceptions of their position in life in the context of the culture and value systems in which they live, and in relation to their goals, expectations, standards, and concerns.”^19^ Within this framework, oral health–related quality of life (OHRQoL) refers to the individual’s subjective evaluation of how oral health affects functional, emotional, and psychological well-being.¹⁶ It encompasses comfort during eating, sleeping, and social interaction, as well as self-esteem and satisfaction with oral health status.^16,20^

A wide range of systemic and localized diseases can negatively influence OHRQoL.^16^ Among these, cancer is particularly detrimental due to the combined burden of the disease itself and the side effects of its often aggressive treatment regimens.^4,5^ Head and neck cancers can directly damage oral tissues or cause functional impairments such as dysphagia, dysarthria, and trismus.^4,5,6,7^ Furthermore, cancer therapies including surgery, radiotherapy, and chemotherapy are frequently associated with debilitating oral complications, including mucositis, xerostomia, dysgeusia, opportunistic infections, and osteoradionecrosis.^4,5,6,7^

Cancer remains a major public health challenge in Saudi Arabia, representing one of the leading causes of morbidity and mortality across all age groups. Pediatric cancers, while relatively rare compared to adult malignancies, impose important physical, psychological, and social burdens on affected children and their families.^12^ In Saudi Arabia, leukemia is the most prevalent pediatric malignancy, particularly among children aged 1–5 years, and continues to be a leading cause of morbidity and mortality in this population.^12^ Other frequently reported childhood cancers include lymphomas (17%), central nervous system (CNS) neoplasms (12%), neuroblastoma (6%), renal tumors (5%), soft-tissue sarcomas (6%), and bone tumors (6%).^12^ Notably, approximately one-third of all pediatric cancer cases nationwide (35%) are managed at King Faisal Specialist Hospital and Research Center (KFSHRC), which serves as the primary national referral and treatment center for childhood malignancies.^2^

Despite the growing cancer burden, only limited research has explored the OHRQoL of pediatric cancer patients in Saudi Arabia. Understanding this relationship is essential, as cancer therapies often cause oral complications that may persist long after treatment, impacting speech, nutrition, and psychosocial well-being. Several instruments have been developed to measure OHRQoL, among which the Oral Health Impact Profile (OHIP) is one of the most extensively validated.^16^ The short-form OHIP-14, employed in this study, consists of 14 items across seven domains: functional limitation, physical discomfort, psychological discomfort, physical disability, psychological disability, social disability, and handicap.^16^

Therefore, this study aimed to assess and explore the factors influencing OHRQoL among pediatric cancer patients and survivors in Saudi Arabia using the OHIP-14 questionnaire. In addition, we sought to describe self-reported oral health practices, access to dental care, and related behaviors in this population. We hypothesized that pediatric cancer patients would exhibit statistically significantly impaired OHRQoL compared to their healthy peers, and that disease- and treatment-related factors would be associated with greater oral health burden.

## MATERIALS AND METHODS 

This study, conducted in collaboration with KFSHRC, employed a hospital-based, questionnaire-driven, cross-sectional design to examine the impact of OHRQoL among pediatric cancer patients.

### Study Population and Data Collection

A total of 100 pediatric patients (aged 1–12 years at diagnosis) were recruited from the Pediatric Oncology Department at King Faisal Specialist Hospital and Research Center (KFSHRC). Eligible participants included children who were currently undergoing, or had completed, surgery, chemotherapy, and/or radiotherapy. Recruitment began in February 2024, and data collection was conducted between February and April 2024.

Participant recruitment followed a purposive (non-probability) sampling approach, given the challenging nature of enrolling this patient population. Sample size calculation was according to data from 2005–2009, a total of 3885 pediatric cancer patients were registered in the Saudi National Cancer Registry (SA-NCR), which operates under a governmental mandate.^2^ Considering that KFSHRC is the largest pediatric cancer treatment center in the country, the estimated target sample size for this study was set at a minimum of 100 participants, assuming that the prevalence of childhood cancer has increased since 2009. The sample size calculation was based on categorical variables, with an assumed margin of error of ±5% and a 90% confidence level.

### Ethical Considerations

Study objectives and procedures were explained to all participants and their parents/guardians by an investigator. Participation was entirely voluntary, with the option to withdraw at any time without repercussions. Written informed consent (from parents/guardians) and assent (from children, when applicable) were obtained prior to data collection, with documents provided in both Arabic and English. Ethical approval was granted by the Institutional Review Board (IRB) at Princess Nourah bint Abdulrahman University (Log Number: 23-0554), along with official collaboration approval from KFSHRC.

### Detailed Questionnaire

The questionnaire was translated into Arabic and back-translated into English by two bilingual investigators to ensure linguistic and conceptual equivalence. Reliability was evaluated through a pilot test involving five participants (excluded from the final analysis), who assessed the clarity and comprehensibility of the questions. Content validity was confirmed by two subject matter experts.

The final questionnaire consisted of 31 items organized into three main sections: 1. demographic and clinical data, extracted from patient medical records and included information on age, gender, medical history, current medications, cancer type, treatment modality, and time since completion of therapy; 2. self-reported oral health practices, collected from participants or their caregivers, covering oral hygiene routines, frequency of dental visits, and reasons for seeking dental care; 3. Oral Health Impact Profile (OHIP-14), a validated 14-item instrument assessing OHRQoL across seven domains: functional limitation, physical discomfort, psychological discomfort, physical disability, psychological disability, social disability, and handicap. Each item was scored on a 5-point Likert scale (0 = never, 1 = hardly ever, 2 = occasionally, 3 = fairly often, 4 = very often/every day). Total scores ranged from 0 to 56, with higher values indicating poorer OHRQoL.

Three investigators, trained and calibrated by the principal investigator (PI) and a senior expert, were responsible for data collection. They gathered demographic and clinical information from patient files and administered the oral health and OHIP-14 components directly to participants through structured face-to-face interviews. Training and calibration were conducted prior to data collection through a pilot study to ensure consistency, accuracy, and inter-examiner reliability. For very young children (<7 years), responses were obtained from parents or guardians as proxy reporters, whereas older children (≥7 years) self-reported with examiner support as needed.

### Analysis

Descriptive statistics (frequencies, percentages, means, and 95% confidence intervals) were used to summarize sociodemographic characteristics and OHIP-14 scores. OHIP-14 item responses were dichotomized into moderate (1–2) and high (≥3), with higher values indicating poorer OHRQoL.

Comparative inferential analyses were performed using the chi-squared test to assess associations between participant characteristics and three outcomes: 1. daily toothbrushing frequency (yes/no); 2. access to dental care within the past 12 months (visited/never); and 3. high OHRQoL impact (OHIP-14 ≥ 3). Statistical significance was set at p < 0.05. All analyses were conducted using Stata 14.2 (Stata; College Station, TX, USA).

## RESULTS

### Demographic Characteristics of Pediatric Cancer Patients at KFSHRC

Among the 100 pediatric cancer patients included in this study, the largest age group was 2–4 years (44%), followed by 8–11 years (22%), 5–7 years (19%), and ≤1 year at the time of diagnosis (15%). Slightly more than half of the patients were male (53%). Most children came from large households, with nearly half (46%) living in families of seven or more members (Table 1).

**Table 1:** Demographic and clinical characteristics of pediatric cancer patients at KFSHRC

Category total	Characteristics	N = 100 (%)	Brushing (no brushing = 30%)	Access to dental care (never = 50%)	OHIP-14 (≥ 3= 52%)
Age	≤1 year	15%	40%	53.3%	40.0%
	2–4 years	44%	27.3%	43.2%	56.8%
	5–7 years	19%	31.6%	52.6%	36.8%
	8–11 years	22%	27.3%	59.1%	63.6%
Sex	Male	53%	41.5%	49.1%	52.8%
	Female	47%	10.2%	51.1%	51.0%
Household size	2–3 members	6%	50.0%	83.3%	83.3%
	4–5 members	29%	27.6%	37.9%	48.3%
	≥6 members	19%	21.1%	42.1%	63.2%
Father’s education	< High school	21%	57.1%	61.9%	57.1%
	< College	26%	13.8%	42.9%	42.9%
	College	38%	18.4%	39.5%	28.6%
	≥ College	6%	16.7%	33.3%	50.0%
Mother’s education	< High school	26%	26.9%	46.2%	65.4%
	< College	9%	22.2%	88.9%	33.3%
	College	40%	25.0%	35.0%	47.5%
	≥ College	25%	44.0%	64.0%	52.0%
Father’s job	Government	29%	24.1%	41.4%	55.2%
	Private	13%	23.1%	46.2%	61.5%
	Military	22%	27.3%	54.6%	45.5%
	Unemployed	9%	33.3%	77.8%	55.6%
	Retired	18%	50.0%	50.0%	44.4%
Mother’s job	Government	26%	26.9%	42.3%	57.7%
	Retired	1%	0.0%	100.0%	100.0%
	Homemaker	42%	28.6%	42.9%	50.0%
Cancer diagnosis	Leukemias/myelo	42%	28.6%	42.9%	50.0%
	Lymphomas	12%	66.7%	75.0%	66.7%
	CNS Tumors	12%	25.0%	41.7%	41.7%
	Peripheral tumors	5%	40.0%	40.0%	80.0%
	Retinoblastomas	10%	30.0%	60.0%	50.0%
	Renal tumors	4%	0.0%	25.0%	75.0%
	Other solid tumors	11%	9.1%	45.5%	45.5%
	Germ cell tumors	1%	0.0%	100.0%	100.0%
	Other/Unspecified	3%	33.3%	100.0%	100.0%
Treatment modality	No treatment	2%	0.0%	50.0%	50.0%
	Chemotherapy only	44%	31.8%	54.6%	40.9%
	Surgery	9%	66.7%	45.5%	66.7%
	Radiotherapy only	1%	0.0%	100.0%	100.0%
	Chemo + surgery	23%	30.4%	43.5%	56.5%
	Chemo + radiotherapy	18%	16.7%	50.0%	66.7%
	Radio + surgery	1%	0.0%	100.0%	100.0%
The total sample size was N = 100, this table only reported percentages.					

Regarding parental education, 38% of fathers and 40% of mothers held a college degree, while 21% of fathers and 25% of mothers had not completed high school. A majority of mothers (72%) were not employed, whereas fathers were most commonly employed in the governmental sector (29%), followed by the military (22%) and private sector (13%). Leukemias, myeloproliferative, and myelodysplastic disorders were the most common diagnoses (42%), followed by lymphomas and reticuloendothelial neoplasms (12%), central nervous system tumors (12%), soft-tissue and other extraosseous sarcomas (11%), and retinoblastomas (10%). Regarding treatment modalities, chemotherapy alone was the most frequently reported (44%), followed by chemotherapy with surgery (23%), combined chemotherapy, surgery, and radiotherapy (14%), and chemotherapy with radiotherapy (12%) (Table 1).

### Access to Oral Healthcare and Oral Health Practices among Pediatric Cancer Patients at KFSHRC

Overall, 30% of pediatric cancer patients reported not brushing their teeth at least once daily. Among these, 41.5% were male and 17% were female. Poor oral hygiene was more prevalent among children whose parents had lower educational attainment; 57.1% of those whose fathers and 44% of those whose mothers had not completed high school did not brush daily. Additionally, 66.7% of patients diagnosed with lymphomas and reticuloendothelial neoplasms reported not brushing daily. With respect to treatment modality, 32% of patients receiving chemotherapy alone and 30.4% of those treated with combined chemotherapy and surgery reported not brushing daily (Table 1).

Regarding access to oral healthcare, half of the patients (50%) had not visited a dentist or dental care provider in the past 12 months. Limited access to dental care was particularly evident among children from larger households (≥7 members, 56.5%). Among patients whose fathers had not completed high school, 62% reported not having had any dental visits, while 61% of those whose fathers had completed only high school also had no dental visits. In terms of diagnosis, 43% of children with leukemias, myeloproliferative, and myelodysplastic diseases and 75% of those with lymphomas and reticuloendothelial neoplasms had not accessed dental care. Similarly, 55% of patients treated with chemotherapy alone and 43.5% of those receiving chemotherapy with surgery had not sought dental services (Table 1).

**Fig 1:**
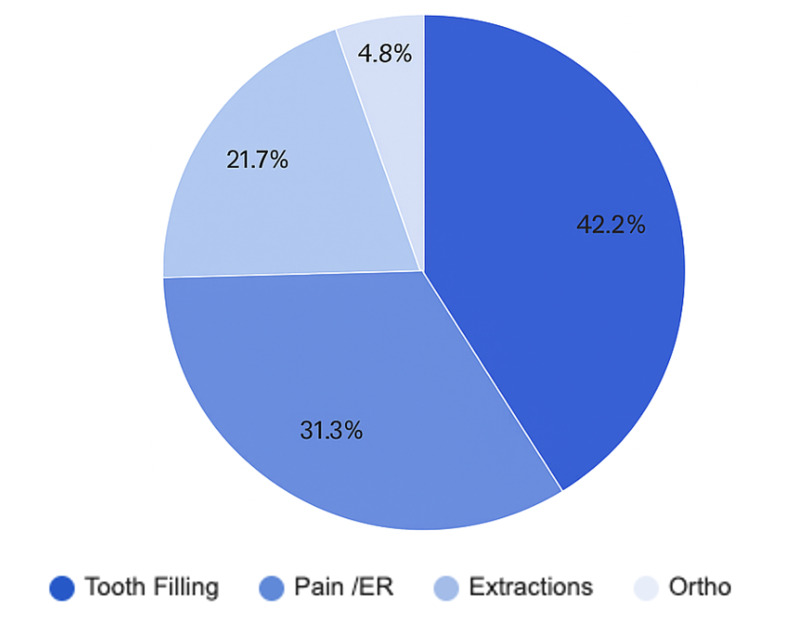
Distribution of reasons for accessing dental care within the past 12 months among pediatric cancer patients KFSHRC.

Among patients who accessed dental care, the most common reason for the visit was restorative treatment (fillings, 42.2%), followed by pain-related or emergency visits (31.1%), extractions (21.7%), and orthodontic care (4.8%) (Fig 1).

### OHRQoL Assessed by the OHIP-14

More than half of the participants (52%) had an OHIP-14 score of ≥3, indicating a considerable impact of oral health on overall quality of life (Table 1).

**Fig 2:**
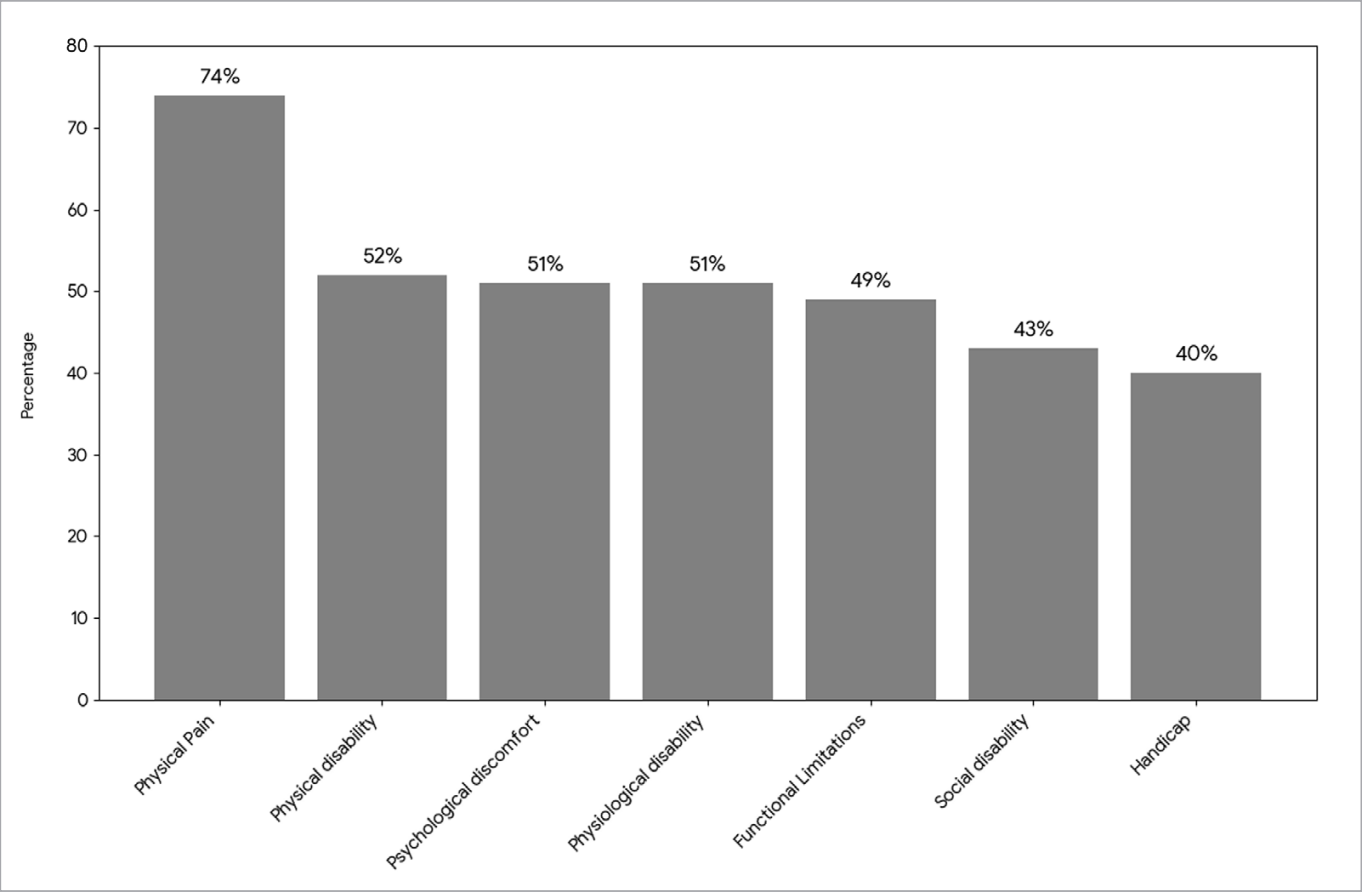
Distribution of high OHIP-14 scores (≥3) across the seven domains among pediatric cancer patients at KFSHRC. The y-axis indicates the percentage of participants.

As shown in Fig 2, high OHIP-14 scores (≥3) were most frequently reported in the physical pain domain (74%), followed by physical disability (52%), while the handicap domain demonstrated the lowest proportion of high scores (40%).

**Fig 3:**
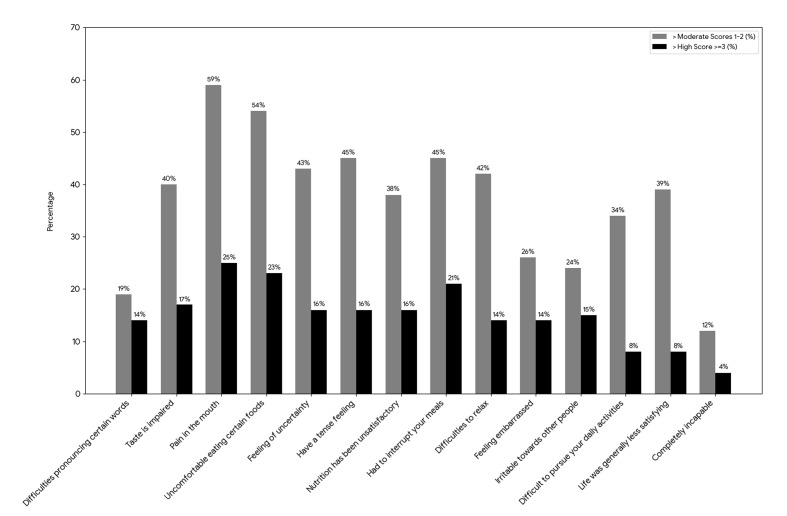
Distribution of OHIP-14 Items (moderate vs. high scores) reported by pediatric cancer patients at KFSHRC. The y-axis indicates the percentage of participants.

Figure 3 illustrates the distribution of participants reporting moderate (scores 1–2) or high (scores ≥3) impacts across individual OHIP-14 items. The highest proportions of moderate scores were observed for “pain in the mouth” (59%), “uncomfortable eating certain foods” (54%), and “feeling of uncertainty” (43%). High scores were most commonly reported for “pain in the mouth” (25%), followed by “uncomfortable eating certain foods” (23%) and “had to interrupt meals” (21%). Conversely, the lowest frequencies of high scores were reported for “life was generally less satisfying” (8%), “difficulty pursuing daily activities” (8%), and “completely incapable” (4%).

Across demographic subgroups, a higher proportion of older children aged 8–11 years (64%) had OHIP-14 scores of ≥3, compared with younger age groups. Among children whose fathers were unemployed, 67% recorded OHIP-14 scores of ≥3, indicating poorer oral health–related quality of life. By diagnosis, elevated OHIP-14 scores were observed among patients with lymphomas and reticuloendothelial neoplasms (67%), followed by those with leukemias, myeloproliferative, and myelodysplastic diseases (50%) (Table 1).

Tables 2 and 3 summarize the mean OHIP-14 domain and item scores with their corresponding 95% confidence intervals (CIs). The highest mean domain score was observed for physical pain (35.81; 95% CI: 26.41–45.20), with “pain in the mouth” (37.0; 95% CI: 29.52–44.47) and “uncomfortable eating certain foods” (32.5; 95% CI: 25.19–39.81) representing the most affected individual items. The functional limitation domain also demonstrated a high mean score (27.4; 95% CI: 18.59–36.21), particularly for impaired taste (24.25; 95% CI: 17.51–30.99). Within the physical disability domain, “had to interrupt meals” had a mean score of 29.5 (95% CI: 21.92–37.07). Psychological discomfort was reflected most in “having a tense feeling” (27.0; 95% CI: 19.93–34.07). In contrast, lower mean scores were recorded in the social disability (17.39; 95% CI: 9.93–24.85) and handicap (8.53; 95% CI: 3.15–13.91) domains, with “completely incapable” being the lowest individual item score (8.5; 95% CI: 3.97–13.02) (Tables 2 and 3).

**Table 2:** Mean high OHIP-14 scores (≥3) among pediatric cancer patients at KFSHRC, by major domain

Domain	OHIP-14 High score (≥3)	Mean (95% CI)
1. Functional limitations	Yes	27.4 (18.59 – 36.21)
2. Physical pain	Yes	35.81 (26.41 – 45.20)
3. Psychological discomfort	Yes	19.83 (12.08 – 27.58)
4. Physical disability	Yes	26.62 (17.94 – 35.29)
5. Physiological disability	Yes	20.52 (12.59 – 28.45)
6. Social disability	Yes	17.39 (9.93 – 24.85)
7. Handicap	Yes	8.53 (3.15 – 13.91)


**Table 3:** Mean OHIP-14 scores (≥1) of pediatric cancer patients at KFSHRC by items and sociodemographic characteristics

OHIP-14 items	Mean (95% CI)	Characteristics		Mean (95% CI)
1. Difficulties pronouncing certain words	14.75 (8.28 – 21.21)	Age	≤1 year	8.67 (2.84 – 14.50)
2. Taste is impaired	24.25 (17.51 – 30.99)		2–4 years	14.77 (10.31 – 19.24)
3. Pain in the mouth	37.00 (29.52 – 44.47)		5–7 years	9.58 (4.63 – 14.52)
4. Uncomfortable eating certain foods	32.50 (25.19 – 39.81)		8–11 years	12.91 (8.21 – 17.61)
5. Feeling of uncertainty	25.25 (18.36 – 32.13)	Sex	Male	13.87 (10.29 – 17.44)
6. Have a tense feeling	27.00 (19.93 – 34.07)		Female	10.87 (7.32 – 14.43)
7. Nutrition has been unsatisfactory	21.50 (15.04 – 27.96)	Household size	2–3 members	16.83 (1.79 – 31.87)
8. Had to interrupt your meals	29.50 (21.92 – 37.07)		4–5 members	14.41 (8.85 – 19.98)
9. Difficulties to relax	22.75 (16.25 – 29.25)		6 members	12.32 (6.55 – 18.08)
10. Feeling embarrassed	17.75 (11.01 – 24.48)		≥7 members	10.72 (7.36 – 14.07)
11. Irritable towards other people	17.50 (10.61 – 24.39)	Diagnosis	Leukemias/myeloproliferative and myelodysplastic	12.93 (8.74 – 17.12)
12. Difficult to pursue daily activities	16.00 (10.47 – 21.53)		Lymphomas and reticuloendothelial neoplasms	16.92 (6.88 – 26.95)
13. Life was generally less satisfying	17.25 (11.76 – 22.74)		CNS and intracranial/ intraspinal neoplasms	8.17 (3.70 – 12.63)
14. Completely incapable	8.50 (3.97 – 13.02)		Retinoblastoma	12.30 (3.14 – 21.46)
			Soft tissue and other sarcomas	9.18 (4.37 – 13.99)
		Treatment	Chemotherapy only	10.02 (6.41 – 13.63)
			Chemo + surgery	16.13 (9.65 – 22.61)
			Chemo + radiotherapy	13.17 (8.34 – 17.99)
			Combination therapy	16.21 (7.86 – 24.57)


### Association Between Participant Characteristics, Oral Health Behaviors, Access to Dental Care, and OHRQoL among Pediatric Cancer Patients 

Chi-squared analyses examined associations between sociodemographic and clinical variables and oral health outcomes. Statistically significant differences in daily brushing frequency were observed by sex and father’s education (p < 0.05 for both) (Table 4). Access to dental care was statistically significantly associated with mother’s education (p < 0.001). No statistically significant associations were found between high OHIP-14 scores and any sociodemographic or clinical variables.

**Table 4:** Association between participant characteristics, oral health behaviors, access to dental care, and oral health–related quality of life among pediatric cancer patients (n = 100)

Variable	Brushing frequency (Yes vs no) χ² (chi-squared)	p-value	Access to dental care (Yes vs no) χ² (chi-squared)	p-value	OHIP-14 (High vs moderate scores) χ² (chi-squared)	p-value
Age	0.97	0.808	1.66	0.645	4.22	0.239
Sex	7.11	**0.008**	0.04	0.841	0.031	0.860
Household size	2.1	0.553	5.61	0.132	4.21	0.235
Father’s education	11.19	**0.025**	4.99	0.290	3.81	0. 431
Mother’s education	3.19	0.364	11.16	**0.011**	3.45	0.328
Father’s job	4.24	0.375	4.01	0.548	5.70	0.336
Mother’s job	0.41	0.816	2.67	0.445	3.35	0.341
Cancer diagnosis	12.55	0.128	9.88	0.273	8.41	0.394
Treatment modality	4.94	0.667	3.37	0.848	6.51	0.482
Chi-squared analyses examined associations between sociodemographic and clinical characteristics with (1) daily toothbrushing frequency, (2) access to dental care within the past 12 months, and (3) oral health–related quality of life (OHIP-14 ≥ 3). Values on bold indicate statistical significance.						

## DISCUSSION

To our knowledge, this is the first study in Saudi Arabia to comprehensively assess self-reported access to oral healthcare, oral hygiene practices, and determinants of OHRQoL among pediatric cancer patients and survivors. In this cohort, leukemias, myeloproliferative, and myelodysplastic disorders were the most common diagnoses (42%), followed by lymphomas and reticuloendothelial neoplasms (12%) and central nervous system tumors (12%). Elevated OHIP-14 scores across multiple domains reflected a considerable burden of oral health–related challenges and their psychosocial consequences in this population. Nearly half of the participants had not received dental care in the preceding year, underscoring an important service gap and highlighting the need to integrate routine dental assessment, preventive counseling, and timely referral into pediatric oncology care pathways.

Statistically significant disparities in oral hygiene practices and access to dental care were observed across sociodemographic and clinical groups. Approximately one-third of the participants did not brush their teeth daily, a behavior more prevalent among male children and those whose parents had lower educational attainment. Parental education, a key indicator of socioeconomic status, is closely linked to health literacy, awareness of oral–systemic connections, and navigation of healthcare services.^7,10,15^ Higher education levels often translate into greater understanding of the importance of maintaining oral hygiene during cancer treatment, better recognition of early oral symptoms, and more proactive engagement with preventive and follow-up dental care. These differences can shape caregivers’ adherence to recommended brushing routines, dietary modifications, and appointment attendance, all of which influence the child’s OHRQoL. Further, poor oral hygiene among pediatric oncology patients has well-documented clinical implications: it increases the risk of oral mucositis, opportunistic infections, caries, and delayed recovery during chemotherapy, particularly in immunocompromised states.^4,6,17^

Interpreted within the contemporary OHRQoL framework encompassing oral function, orofacial pain, orofacial appearance, and psychosocial impact,^11^ our findings indicate a substantial multidimensional burden. More than one-third of participants reported severe mouth pain (37%) and difficulty eating (33%), reflecting the direct impact of therapy-related oral complications, such as mucositis, xerostomia, and altered taste, on essential daily functions like nutrition and speech.^1,4^ These functional limitations often extend beyond physical discomfort, contributing to psychological distress and social withdrawal. Moreover, approximately half of the participants expressed general life dissatisfaction, underscoring the interconnectedness between oral health and broader health-related quality of life (HRQoL) domains, where physical symptoms translate into emotional and social challenges.

Educational interventions may help address these gaps. Evidence indicates that structured oral health education and preventive programs can significantly improve dental and periodontal outcomes, foster better communication between families and healthcare providers, and enhance caregiver knowledge and engagement, ultimately leading to improved oral hygiene and overall oral health during cancer therapy.¹⁴

This cross-sectional study has several limitations. Causality cannot be inferred, and the modest sample size and nonprobability sampling used limits the precision and generalizability of the findings. Although the 2009 national registry data used are the most recent and only available national reference for this population, this constraint may limit the precision of our sampling justification. Recall bias is also possible, as some survivors were recruited several years post-treatment. Additionally, selection bias may have occurred if participants experiencing oral health problems were more likely to take part in the study. Strengths of this study include its focus on an understudied and vulnerable Saudi pediatric oncology population, the use of a validated OHRQoL instrument (OHIP-14), and the simultaneous evaluation of oral health access, practices, and diagnostic categories. Future research should employ multicenter longitudinal cohorts following patients from diagnosis through survivorship, linking self-reported OHRQoL data with clinical oral examinations to capture objective and subjective dimensions of oral health. Moreover, studies should test integrated care models that embed routine dental screening, referral pathways, and preventive care bundles within oncology services. Interventional trials targeting caregiver- and patient-focused oral health literacy, alongside evaluations of structural barriers such as insurance coverage, scheduling constraints, and workforce distribution, are also warranted. These priorities align with Saudi Vision 2030’s Quality of Life Program, which emphasizes prevention, equitable access, and coordinated, person-centered care.^18^

## CONCLUSION

This study among pediatric oncology patients and survivors highlights insufficient access to and use of oral healthcare and a notably poor OHRQoL. The findings underscore the need to integrate routine oral screening, early dental referral, and preventive care within pediatric oncology care pathways, supported by stronger provider coordination and family-centered health literacy initiatives. Policy-level interventions should aim to mitigate structural barriers such as inadequate insurance coverage, limited appointment availability, and travel constraints. Future multicenter, longitudinal studies that combine OHIP-14 assessments with clinical oral evaluations are warranted to identify modifiable risk factors and to evaluate integrated care models capable of improving both oral and overall quality of life outcomes in this vulnerable population.

## ACKNOWLEDGEMENTS

Princess Nourah bint Abdulrahman University Researchers Supporting Project number (PNURSP2026R389), Princess Nourah bint Abdulrahman University, Riyadh, Saudi Arabia. Additionally, the authors gratefully acknowledge the support of King Faisal Specialist Hospital and Research Center (KFSH&RC) for their support of this study. Special thanks are extended to Amani AlKofide, MD, Viqaruddin Mohammed and Professor Mamata Hebbal for their valuable support.
